# Supplementary material for: Proteomic characterization and comparison of ram (Ovis aries) and buck (Capra hircus) spermatozoa proteome using a data independent acquisition mass spectometry (DIA-MS) approach
Source: PLoS One. 2020 Feb 13;15(2):e0228656. doi: 10.1371/journal.pone.0228656 (PMC7018057; doi:10.1371/journal.pone.0228656)
Supplement: Attachment — Submitted filename: Response to Reviewers'.docx [file pone.0228656.s010.docx]

**Responses to the comments by Editor and Reviewers**

Dear Editor and Reviewers:

Thank you very much for your and the reviewers’ comments concerning our manuscript entitled “Proteomic characterization and comparison of spermatozoa proteomes between ram (*Ovis. aries*) and buck (*Capra. hircus*) using a data-independent acquisition-mass spectrometry (DIA-MS)” (No. PONE-D-19-15817R1). These comments are all valuable and very helpful for improving our paper. We have studied comments carefully and have made correction and revisions which was yellow-highlighted in the revised version. We have tried our best to revise our manuscript and believe that the revised article has been improved substantially, and hope all these emendations and clarifications will be satisfactory.

Below is point-wise reply to the comments of the Reviewers. Should you need additional information, I shall be happy to provide.

Best regards,

Zi-Jun Zhang

On behalf of all authors

**List of Corrections Made with the Comments of Reviewers**

**Reviewer #1 comments**:

-The authors have addressed almost all the modifications suggested. However, in the present version of the manuscript there are some aspects that still need to be improved.

**AU:** Thanks for your valuable comments on our work, and we have studied comments carefully and have made correction and revisions which was yellow-highlighted in the revised version.

-The explanation of the experimental design is still very poor and although the figure 1 can help, in my opinion is not enough.

**AU:** The explanation of the experimental design has been revised. The explanation of the experimental design was revised as follows: the experimental design and workflow are shown in Fig 1. Semen samples were collected from Hu-sheep rams (n = 9) and Anhui white goat bucks (n = 9). Individual spermatozoa of each species were equally pooled into three biological samples.

-In the current version of the manuscript I have not found the figure legends for the figures provided.
-Only legends for figure 1 and 2 are provided in the main text of the manuscript. This is nor the appropriate place and, in addition, the explanations are not adequate being poor and not informative enough.

**AU:** All figure legends has been revised and shown in the revised version of the manuscript. According to the guide-for-authors, each figure caption should appear directly after the paragraph in which they are first cited. So, all the figure legends were put in in the main text of the manuscript.

-The quality of some figures is still not good, as for example figure 1 and 2. Please, revise it again and try to improve if possible.

**AU:** Revised as you suggested. Resolution of figure 1 and 2 have been improved, and all figures have been uploaded to the Preflight Analysis and Conversion Engine (PACE) digital diagnostic tool to ensure that figures meet PLOS requirements. However, it is strange that the uploaded figures are very clear, but the ones presented in the draft are not good.

-Finally, I recommend the publication of this manuscript after minor revision.
**AU:** Revised as you suggested.

Once again, thank you very much for your comments and suggestions.

**Reviewer #2 comments**:

The Authors have complied with most of my remarks which led to the improvement of the overall quality and sound of the paper. Therefore, I can now recommend the MS for publication.

**AU:** Thank you very much for your positive and valuable comments on our work.
